# Computation of the Retentive Forces of Ball and Magnet Attachments in a Two-Implant-Retained Mandibular Overdenture

**DOI:** 10.7759/cureus.84831

**Published:** 2025-05-26

**Authors:** Sourabh Khandelwal, Oshin Sharma, Rajeev Srivastava, Vivek Choukse, Garima Mukhopadhyay, Miral Mehta, Santosh Kumar

**Affiliations:** 1 Department of Prosthodontics and Crown & Bridge, Index Institute of Dental Sciences, Indore, IND; 2 Department of Orthodontics and Dentofacial Orthopedics, Government Autonomous College of Dentistry, Indore, IND; 3 Department of Prosthodontics and Crown &amp; Bridge, Dr. Hedgewar Smruti Rugna Seva Mandal's Dental College and Hospital, Hingoli, IND; 4 Department of Prosthodontics, Modern Dental College and Research Center, Indore, IND; 5 Department of Pedodontics and Preventive Dentistry, Karnavati School of Dentistry, Karnavati University, Gandhinagar, IND; 6 Department of Periodontology and Implantology, Karnavati School of Dentistry, Karnavati University, Gandhinagar, IND

**Keywords:** ball attachment, denture retention, implant-supported overdenture, magnetic attachment, mandibular overdenture

## Abstract

Background: Advancements in tooth replacement have led to increased use of implant overdentures, prompting the proposal of various attachment systems. Unsplinted options like ball, magnetic, and telescopic attachments offer different retention methods. Ball attachments are straightforward but require periodic O-ring replacement and are limited by implant angulation. In contrast, magnet attachments are versatile and compact, thanks to rare-earth element alloys. Their strength and corrosion resistance make them advantageous.

Objective: This study compares magnetic and ball attachments' retention in mandibular implant overdentures, focusing on B and D implant positions. It aims to determine the most effective attachment for stability and ease of use.

Methods: In this in vivo comparative study, eight completely edentulous patients were selected randomly according to inclusion and exclusion criteria. Two-piece implants were placed in the mandibular arch at the B and D positions. After osseointegration, second-stage surgery was done, and a gingival former was placed. Two mandibular record bases were fabricated for each patient using heat-cure acrylic resin. One for ball attachment (Group A) and the other for magnet attachment (Group B), and retention was measured using a custom-made device. This device allows us to apply an increasing, vertical force on the record base. The force was increased gradually until dislodgement of the denture base occurred. The procedure was repeated three times for all eight patients with ball and magnet attachments. The applied vertical force was registered in kilograms (kg) by the weighing scale, and afterwards, it was expressed in newton (N).

Result: The retention of ball attachment (Group A) was comparatively greater than that of the magnet attachment (Group B).

Conclusion: The retention of mandibular implant-retained overdenture by ball attachments (Group A) was found to be significantly more than that of magnet attachment (Group B).

## Introduction

In an age marked by progress and increasing community awareness regarding tooth replacement, there has been a noticeable rise in cases involving implant overdentures. Consequently, various attachment systems, each with distinct retentive properties, have been proposed for supporting these overdentures [1]. Among these, unsplinted attachment systems have gained widespread popularity, particularly in mandibular implant overdentures, and encompass ball, magnetic, and telescopic attachments [2]. These systems utilize mechanical interlocking, frictional contact, or magnetic forces to achieve retention between the matrix and patrix parts of the attachments [3].

Ball attachments are considered the simplest for clinical application in implant-supported overdentures. However, a primary limitation of this system is the gradual loss of retention with O-rings, necessitating periodic replacement. Stud attachments cannot be employed in cases where implants are non-parallel or have angulations exceeding 15° [4,5].

In contrast, magnet attachments offer versatility, as they can be used with non-parallel implants and require less inter-arch space due to their shorter length compared to mechanical attachments [6].

Recent advancements have seen the use of rare earth element alloys such as samarium and neodymium, which result in magnets significantly stronger than conventional ferrite or alnico alloy magnets [7,8]. Incorporating neodymium/boron/iron alloys in magnet fabrication has notably enhanced their retentive properties [9]. Additionally, stainless steel housing prevents tarnishing and corrosion of magnets from intraoral saliva [10,11].

When selecting attachments for implant-supported overdentures, it is crucial to prioritize sufficient retentive characteristics for improved prosthesis stability while ensuring ease of placement and removal for the patient [12].

This study particularly highlights magnetic attachments, despite their underutilization, due to their advantages. It compares the retention provided by magnetic attachments with ball attachments concerning the retentive force they offer. The study focuses on cases where implants are positioned at B and D locations, deemed most favorable for two-implant retained mandibular overdentures. Thus, the study aims to compare retention in mandibular implant-retained overdentures using ball and magnet attachments at B and D positions in the edentulous mandible.

## Materials and methods

This study was performed in the Department of Prosthodontics at our institute. The institutional review board reviewed and approved the study in March 2022 (IDIRB/2022/03/12). This study was carried out from June 2022 to June 2023. It was done according to the Declaration of Helsinki. The sample size (n) was calculated using the formula "$n\quad =\quad \frac{\left( Z_{\alpha}\quad +\quad Z_{\beta} \right)^{2} \times \quad \sigma^{2}}{d^{2}} \quad$" where SD = anticipated standard deviation = 10. Za = value of Z when a = 5% =1.96. d = minimum expected mean difference = 4. The total sample size calculated was eight. Eight edentulous patients who met the inclusion and exclusion criteria for the study were selected. The group consisted of six male and two female subjects aged 45 to 70 years.

The inclusion criteria for this study encompass patients who are completely edentulous in both the maxillary and mandibular ridges, ensuring a full absence of teeth in these regions. Additionally, patients must possess sufficient residual alveolar ridge structure to accommodate implant placement, ensuring the procedure's feasibility. Adequate inter-arch distance is also required, ensuring enough space between the upper and lower dental arches to accommodate the proposed implant-supported overdenture. These criteria collectively ensure that patients selected for the study are suitable candidates for evaluating implant attachment systems and their impact on prosthesis stability and function.

Patients with systemic diseases such as diabetes mellitus or hypertension, which directly impact bone metabolism and healing, are excluded from the study to ensure that the results are not confounded by these conditions. Likewise, individuals with impacted teeth in the study area, psychological or psychiatric conditions, or developmental malformations of the mandible, such as cysts or tumors, are excluded to maintain homogeneity within the sample population. Patients requiring bone augmentation or presenting with undercuts in the mandibular residual alveolar ridge are also excluded, as these factors could significantly affect the implant procedure and subsequent outcomes. These exclusion criteria help ensure that the study focuses on a specific patient cohort most suitable for evaluating the effectiveness of implant attachment systems.

Preoperative investigation and procedure

**Figure 1:**
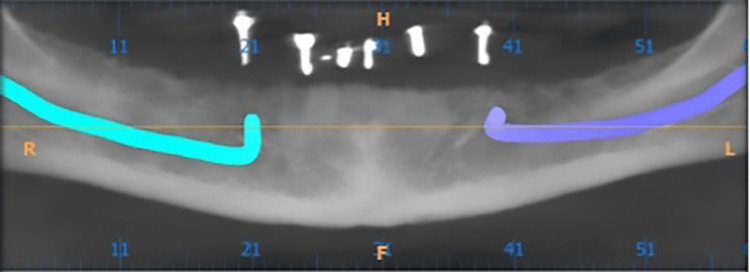
Cone beam computed tomography (CBCT) with radiographic marker. Image Credit: Sourabh Khandelwal

Patients included in the study underwent blood investigations, including complete blood count, random blood sugar, HIV screening, and HBsAg test. Furthermore, a preliminary step of orthopantomography (OPG) was done for all eight patients included in the study for bone height appraisal. Subsequently, following the principles of the impressions of complete denture, the impression was made using impression compound, and through that, a diagnostic cast was made. Then, to estimate the adequate inter-ridge distance for placement of the attachment (ball and magnet) over the implants, diagnostic mounting was done on Hanau wide vue semi-adjustable articulator, and following that, a diagnostic stent was made using acrylic resin, and markings were made on it. A radiographic marker (gutta percha) was placed at A, B, C, D, and E positions, and cone beam computed tomography (CBCT) was performed with the diagnostic stent to measure the height and width of the bone in the inter-foraminal region (B and D position) for the placement of the implants for overdenture (Figure 1).

Surgical procedure

**Figure 2:**
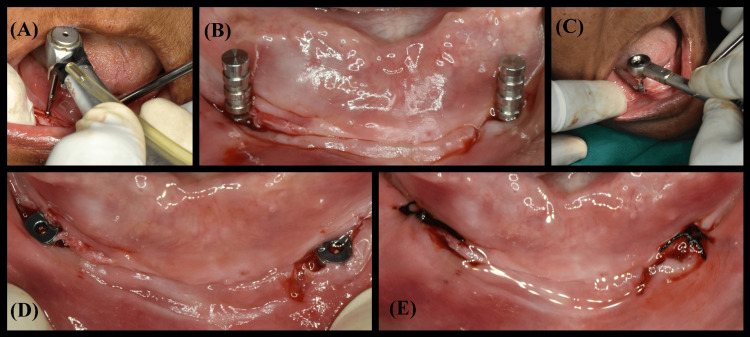
Surgical procedure for implant placement. (A) Marking drill for implant. (B) Guide pins are used to see the implant's parallelism before implant placement. (C) Desired torque achieved. (D) Implant in position. (E) Sutures given. Image Credit: Sourabh Khandelwal

**Figure 3:**
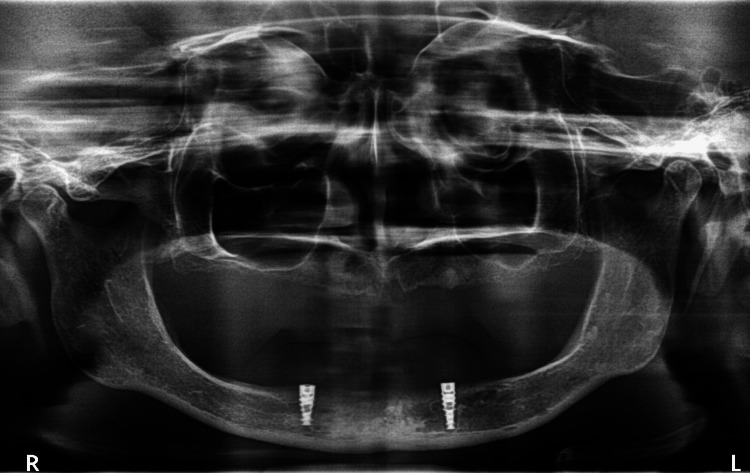
Postoperative orthopantomograph showing the placement of implants. Image Credit: Sourabh Khandelwal

**Figure 4:**
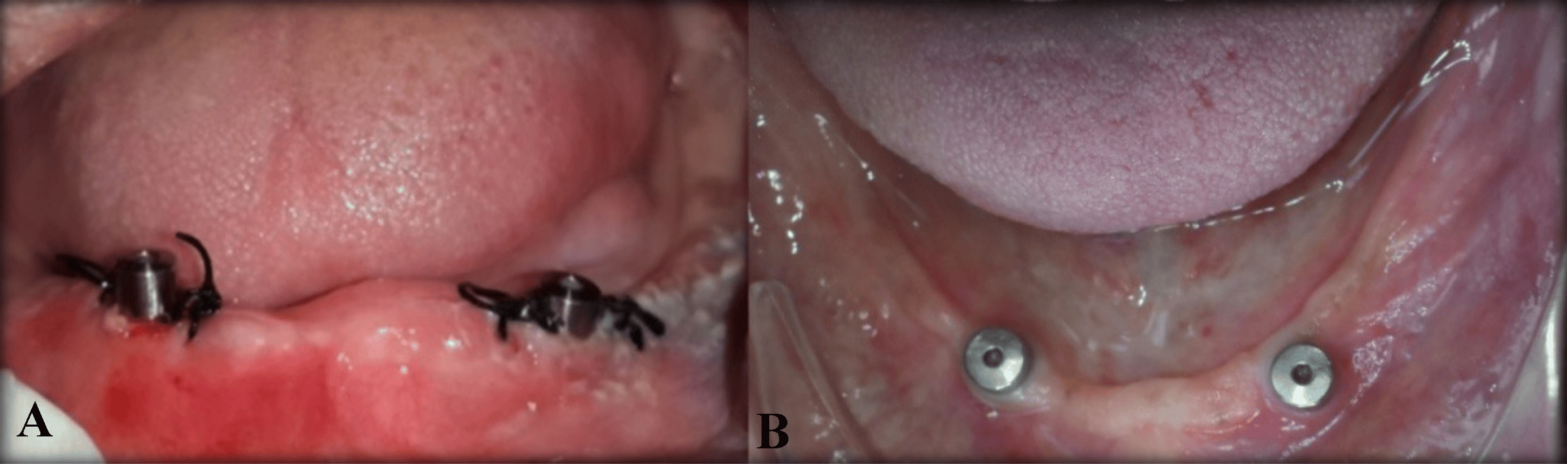
Second-stage surgery. (A) Gingival former placed and suturing done. (B) After healing. Image Credit: Sourabh Khandelwal

According to the standardized surgical protocol for implant placement (Figure 2), each patient received two implants according to the desired dimension of bone in the anterior part of the mandible (at B and D position) between the mental foramina (Figure 3) and follow-up was done every month till three months. After three months of follow-up, osseointegrated implants were inspected, and second-stage surgery was performed with two small crestal incisions at the location of the implants. The implants were exposed, and two gingival formers (Noris Medical, Israel) replaced the two-cover screw (Figure 4).

Prosthetic procedure

The prosthetic procedure was started after the healing of the second stage of surgery. The primary impression of the mandibular arch was made with an impression compound to get a primary mandibular cast. A wax spacer was adapted on this primary cast, and a special tray was fabricated using cold-cure acrylic resin for border molding. Sectional border molding with green stick compound was performed, and relief holes were created at the implant region. The final impression of the mandibular arch was taken with polyvinyl siloxane impression material. The final impression is poured with the die stone to acquire a master cast.

For each patient, two record bases of heat-cured acrylic resin were fabricated, one for ball attachment and the other for magnet attachment. Therefore, the master cast was duplicated using reversible hydrocolloid impression material (agar-agar). During the fabrication of the heat-cured acrylic resin record base, adequate space was provided in the intaglio surface for ball and magnet attachment. 

**Figure 5:**
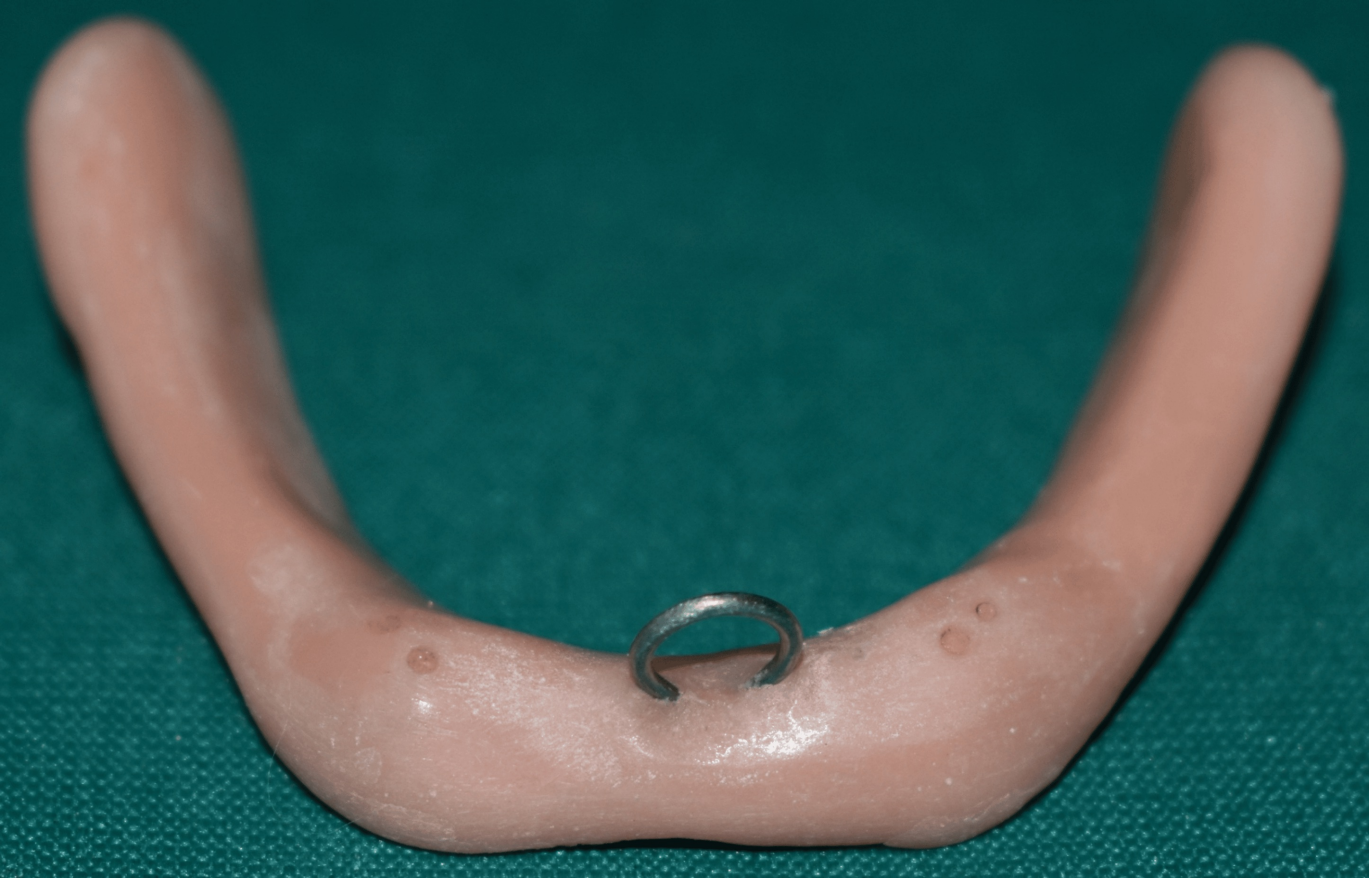
Heat-cure record base showing stainless steel loop attached to the polished surface of record base. Image Credit: Sourabh Khandelwal

Loops were fabricated using 19-gauge stainless steel wire. These loops were attached to the center of the mandibular record base with self-cure acrylic resin material (Figure 5), which was later used to engage the record base to measure the retention of ball and magnet attachment. Petropoulos and Smith [13], Chung et al. [14], Rutkunas et al. [15], Wahab and Sadig [16], Sadig [17], and Scherer et al. [18] have done a study using loops in different positions. In the present study, the loops are attached to the center of the record base.

**Figure 6:**
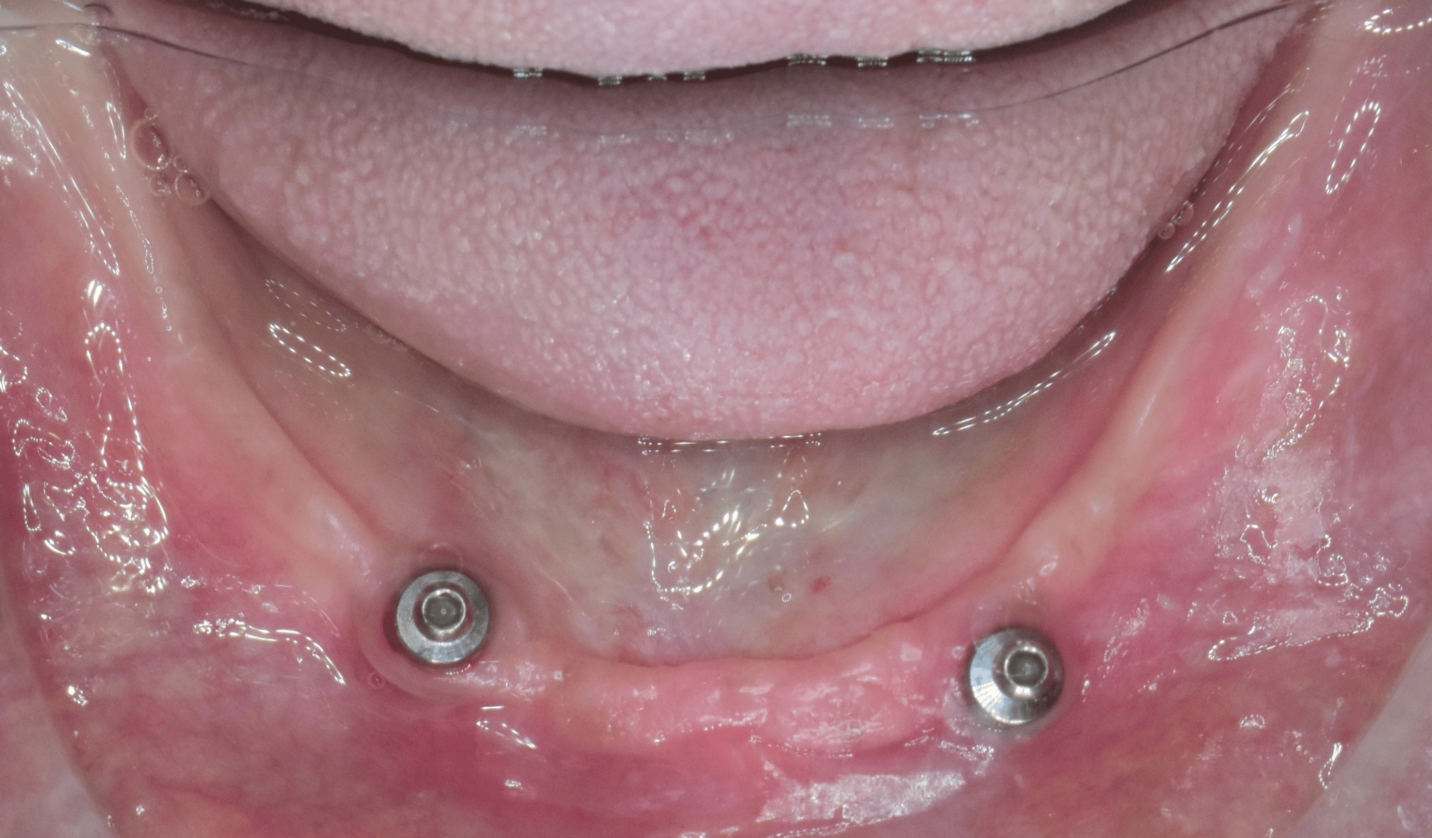
Ball attachment placed into the implants. Image Credit: Sourabh Khandelwal

**Figure 7:**
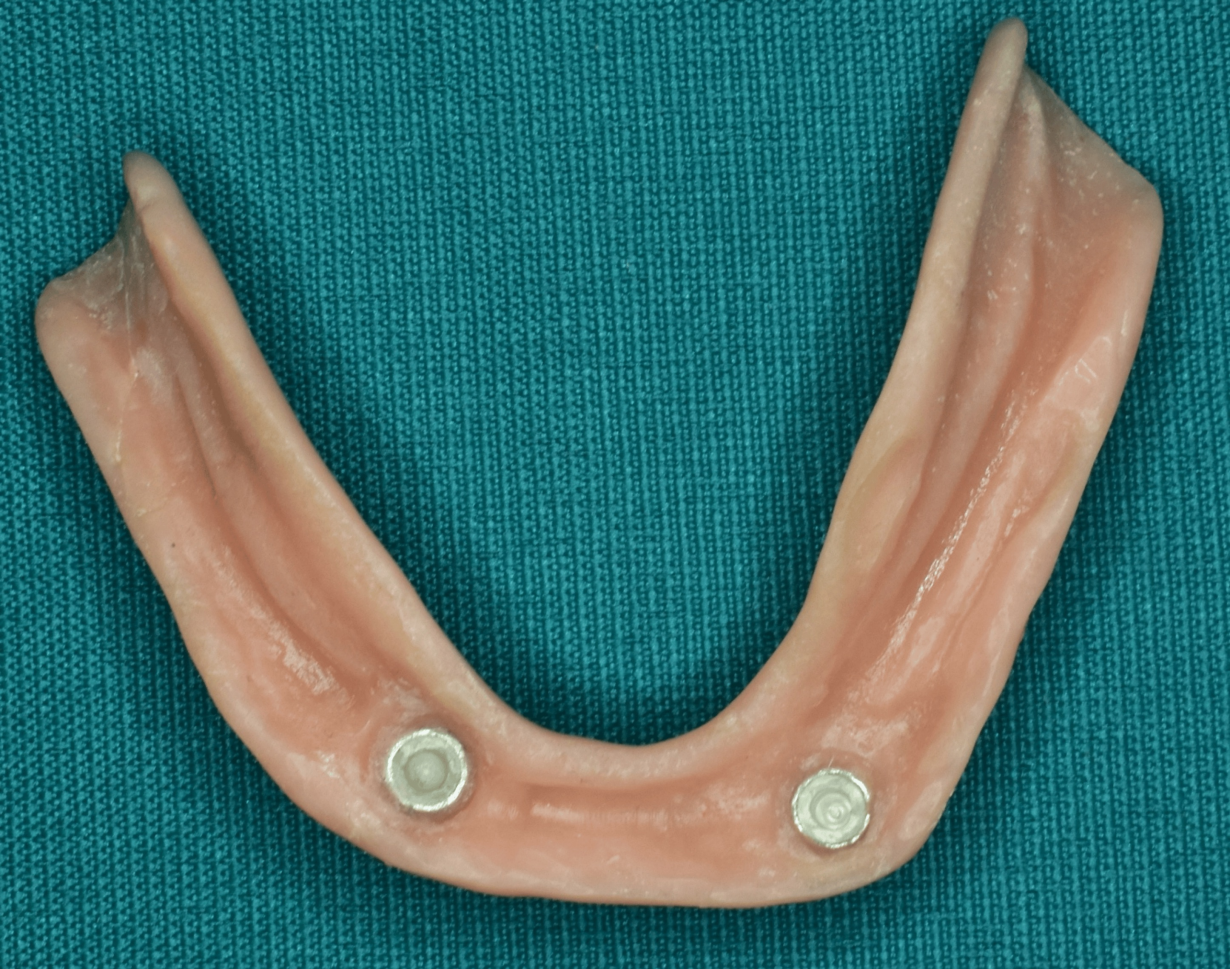
The intaglio surface of the record base shows the picked-up metal housing of the ball attachment. Image Credit: Sourabh Khandelwal

Then, each gingival former was removed from the implant, a ball attachment (Noris Medical, Israel) was placed into the implant (Figure 6), and a metal housing was placed on the ball attachment. Then, the first record base was placed over the mandibular ridge, which contained a ball attachment with metal housings. As a result, on the removal of the record base, the metal housings were picked up in the space created in the intaglio surface of the record base and were secured using self-cure acrylic resin (Figure 7).

**Figure 8:**
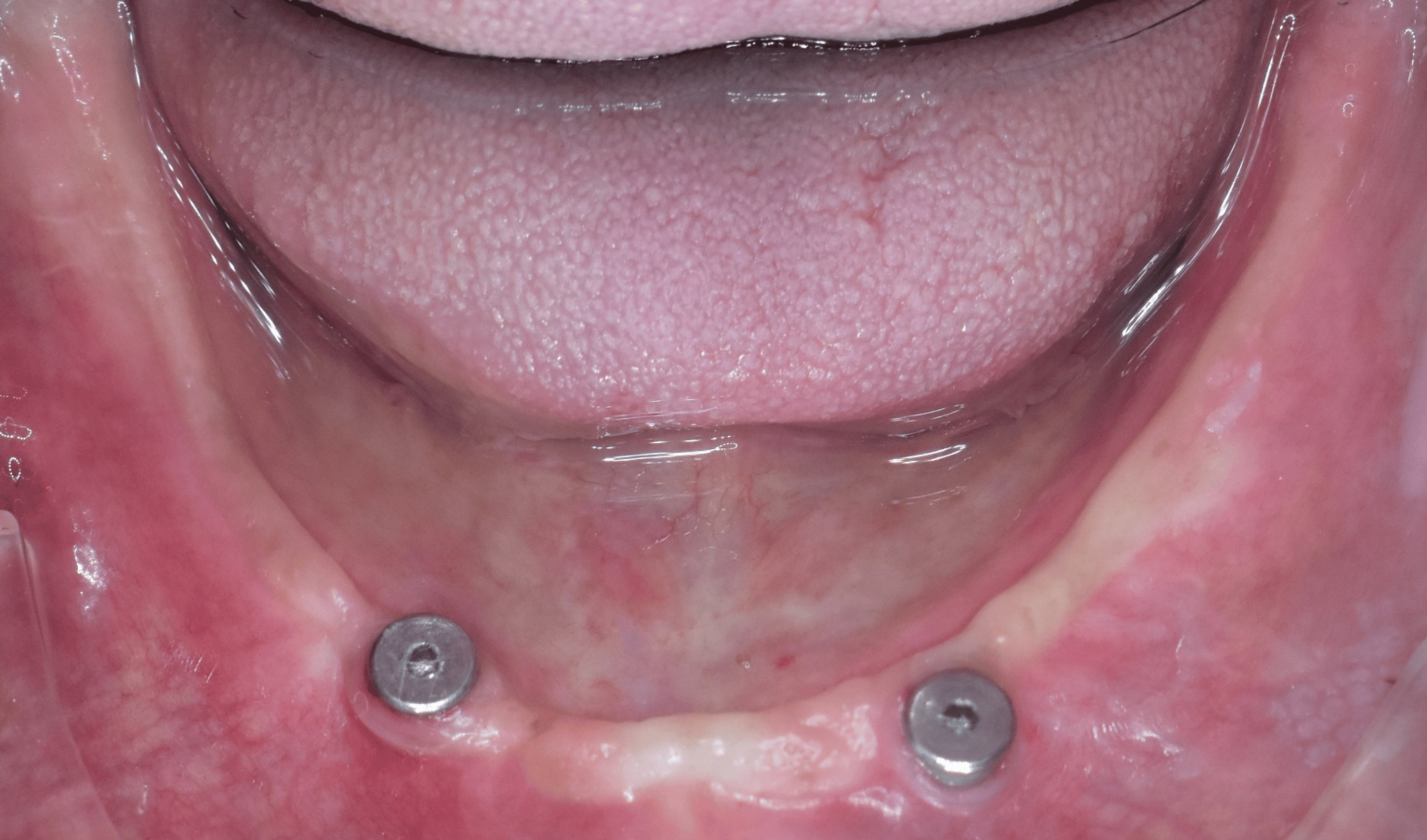
Magnetic keeper placed into the implants. Image Credit: Sourabh Khandelwal

**Figure 9:**
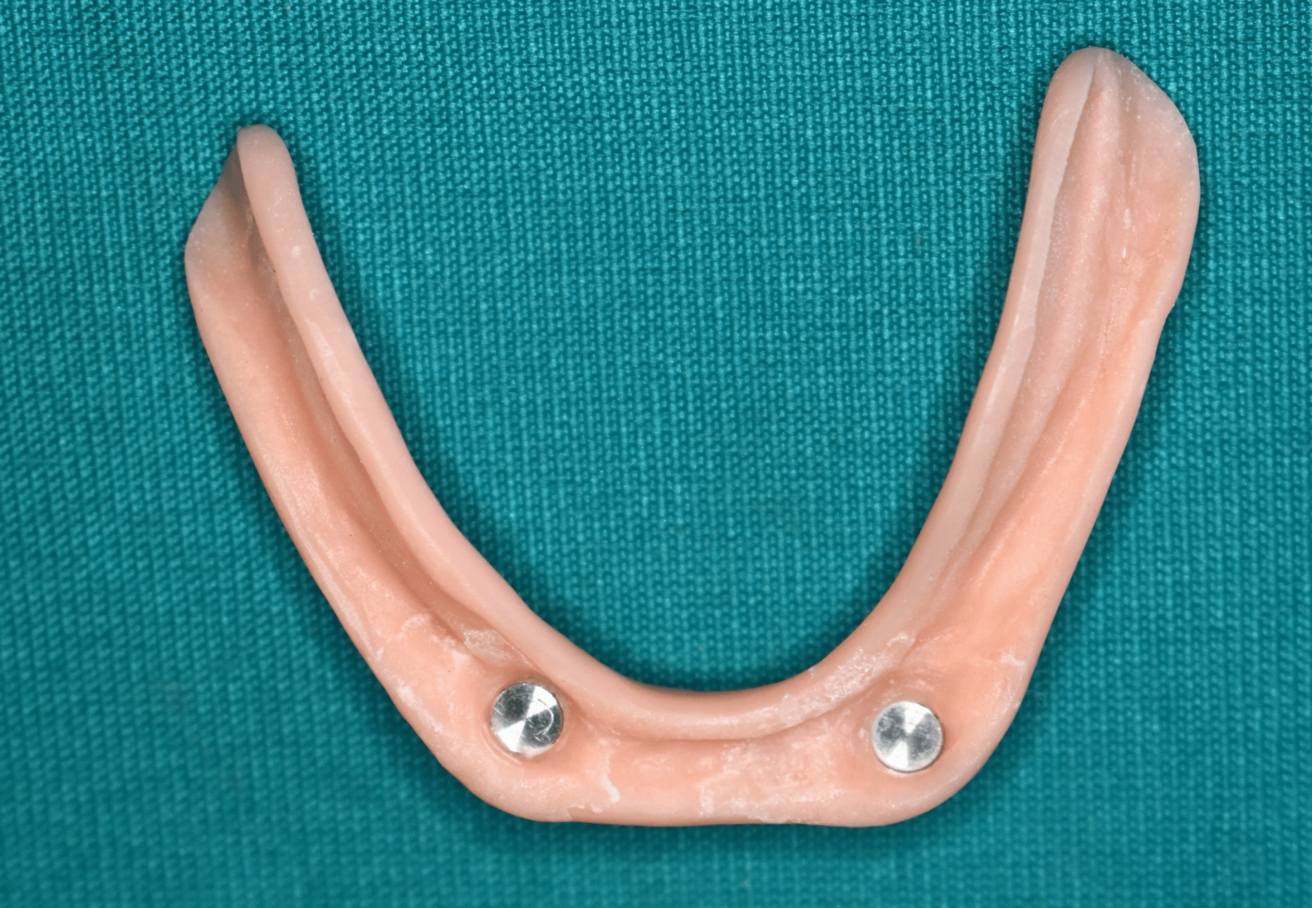
Intaglio surface of record base showing picked-up magnets of magnetic attachment. Image Credit: Sourabh Khandelwal

After that, the ball attachment was removed from the implant, and a magnetic keeper (Dyna, Netherlands) was placed into the implant (Figure 8), and a magnet (Dyna, Netherlands) was placed on the magnetic keeper. Then, the second record base was seated over the mandibular ridge, containing a magnetic keeper magnet. As a result, on the removal of the record base, the magnets were picked up in the space created in the intaglio surface of the record base and were secured using self-cure acrylic resin (Figure 9).

Retention measuring device

**Figure 10:**
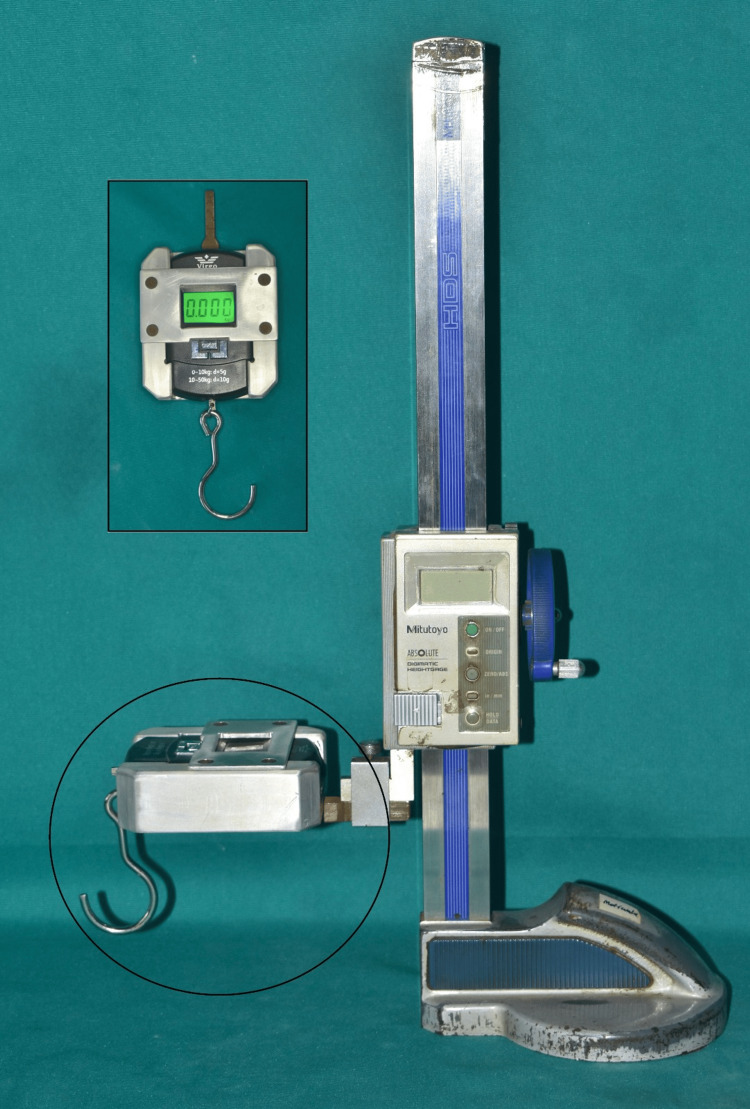
Customized device with weighing scale, hook type, for retention measurement. Image Credit: Sourabh Khandelwal

A device was developed to apply an increasing vertical force on the heat-cure acrylic resin record base. The force was administered through a stainless steel 19-gauge wire fitted with a weighing scale, hook type (weighing) (Figure 10), on one side, and the other was engaged to the loop on the mandibular record base.

Retention measurement

**Figure 11:**
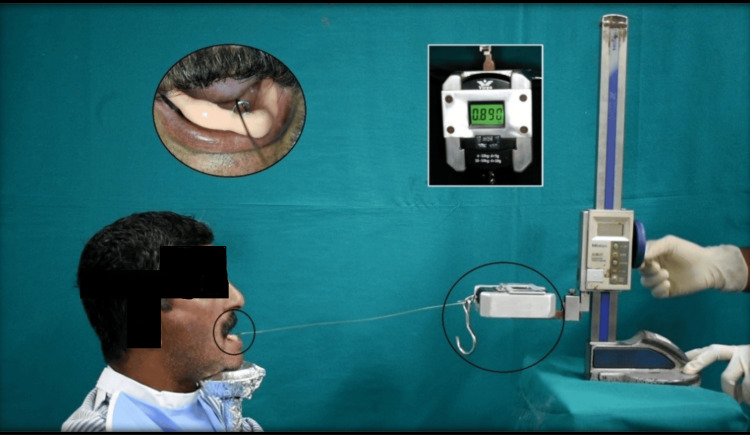
Reading is obtained as the record base is dislodged. Image Credit: Sourabh Khandelwal

The patient was instructed to keep his chin firmly on the chin support. The first record base with metal housing in it was placed over a ball attachment that was placed into the implants, and then a horizontal load frame, which was driven manually, induced the increased vertical force. The force was increased gradually until the dislodgement of the record base occurred (Figure 11). The procedure was repeated three times for all eight patients with ball attachment.

The applied vertical force was registered in kilograms (kg) by the weighing scale, and afterwards, it was expressed in newtons (N). After that, the ball attachment was removed. The second record base with magnets was placed over the magnetic keeper that was placed into the implants, and the above-mentioned process was performed with magnet attachment until dislodgement of the record base occurred. The procedure was repeated three times for all eight patients with magnet attachment.

The readings obtained were then subjected to statistical analysis.

Statistical analysis

The mean values of the two groups were compared using an independent ‘t’ test for two sample means. Data was expressed as mean ± SD. Nonparametric values were compared using the Whitney U or Z tests for two-sample proportions. A p-value of <0.05 was considered significant. SPSS version 22 (IBM Corp., Armonk, NY) was used for statistical calculations.

## Results

The present study used two different attachments to compare the retention in mandibular implant-retained overdenture at B and D positions. The attachments used were ball attachment (Group A) and magnet attachment (Group B). The constraint required to eject the record base from the attachment was registered in kilograms and expressed in newtons (N). Three readings were recorded for each patient to minimize the error with both attachments, and the mean was calculated.

The mean values for retention of ball attachment (Group A) were in the range from 7.91 N to 8.84 N, while those for magnet attachment (Group B) were in the range from 5.07 N to 5.74 N, respectively (Table 1).

**Table 1:** Mean values for retention of mandibular implant-retained overdenture achieved by ball attachment (Group A) and magnet attachment (Group B) in newton (N)

S. No.	Mean value for retention of attachment in newton (N)	
	Ball attachment	Magnet attachment
1.	7.96	5.13
2.	8.07	5.42
3.	7.91	5.26
4.	8.84	5.22
5.	8.13	5.70
6.	8.11	5.07
7.	8.43	5.74
8.	8.78	5.41

The difference in retention among both groups in Newton was compared by using the t-test. The mean of retention for ball attachment (Group A) was 8.27 ± 0.36 N, whereas for magnet attachment (Group B) it was 5.36 ± 0.24 N. The result demonstrated a significant difference between the two groups, with a t-value of 18.30 and a p-value of 0.01 (Table 2). The retention of ball attachment (Group A) was comparatively greater than that of magnet attachment (Group B). This suggests that the ball attachment (Group A) had better retention than the magnet attachment (Group B).

**Table 2:** Comparison of the mean value of retention achieved by ball attachment (Group A) and magnet attachment (Group B) in newton (N) *Significant according to t-test analysis.

	Mean	Standard deviation	t-Value	p-Value
Group A	8.27	0.36	18.30	0.01*
Group B	5.36	0.24		

## Discussion

Nowadays, dental implants are preferred for replacing tooth loss, and two-implant-retained mandibular overdentures are considered the most effective treatment for edentulous mandibles. Different attachment systems have been introduced for implant overdenture, like balls, flats, and magnets. The studies have demonstrated that the retentive force of magnets is sufficient for denture retention and provides great satisfaction to the patient. Magnetic attachments are shorter, especially used in cases of reduced interarch space or moderately non-parallel abutments or patients with physical disabilities, for they are easy to place and remove. The clinical study of Ellis et al. [19] indicated that more than 30% of patients prefer the magnetic attachment as the retention system within implant-supported mandibular overdentures for a comfortable feeling and ease of cleaning. Meanwhile, Cheng et al. [20] showed that implant-retained magnetic attachment can significantly improve mandibular overdenture's masticatory efficiency, comfort level, and satisfaction.

Nevertheless, there are very few studies on the comparison of retentive force between ball and magnet attachment. Moreover, retention and stability are prime factors for implant-supported overdenture. Hence, in this study, retention was measured, which was obtained and compared for both ball and magnet attachments at B and D positions in a mandibular two-implant-retained overdenture.

The device was customized like that in Kampen et al. [21] for evaluation of retention force by both attachment systems. This device applies an increasing vertical force on the heat-cure acrylic resin record base. The force was administered through a stainless steel 19-gauge wire fitted with a weighing scale, hook type (weighing), which measures force in the range of 0.1 kg to 50 kg. The applied vertical force was registered in kilograms (kg) by the weighing scale, and afterwards, it was expressed in newton (N).

In the present study, the retention of ball attachment (Group A) was in the range from 7.91 N to 8.84 N with a mean of 8.27 ± 0.36 N. Tokuhisa et al. [22] found that the ball attachment system is an advantageous attachment system for implant-supported overdenture about optimizing stress and minimizing denture movement. Naert et al. [23] observed that the highest retention force value was shown by ball attachment, i.e., 1,327 g at year 10, compared to bars (1,067 g) and magnets (219 g). Michelinakis et al. [24] observed the highest retention force of 40 N for ball attachments at 29 mm compared to yellow clips, white clips, and magnets. Alsabeeha et al. [25] found that ball attachments achieved higher retention values than magnetic attachments under axially or paraxially dislodging forces. Scherer et al. [18] observed the highest retention value of 37.17 N for ball attachment, followed by locator (pink), O-Ring, and ERA in a vertically directed test.

In the present study, the retention of magnet attachment (Group B) ranged from 5.07 N to 5.74 N with a mean of 5.36 ± 0.27 N. Similar results were observed, respectively. Rutkunas et al. [26] also noticed less retentive and stabilizing forces for magnetic attachment, i.e., 0.46 to 2.79 N, with all types of dislodgement compared to stud attachments with the retentive value of 2.78 to 13.27 N. Naert et al. [27] after five years of observation concluded that bar attachment system had the highest retention value of 1240 g and had least prosthetic complications. Ball attachment remained more stable with a retention value of 567 g, while magnet attachment showed a lower retention value of 110 g. Whereas, Boeckler et al. [28] noted that maximum retention forces shown by magnetic attachments on implants ranged from 0.7 to 5.8 N, and the maximum retention force for root keepers ranged from 1.4 to 6.6 N.

In the present study, the retention of ball attachment (Group A) was in the range from 7.91 N to 8.84 N with a mean of 8.27 ± 0.36 N, while the retention of magnet attachment (Group B) was in the range from 5.07 N to 5.74 N with a mean of 5.36 ± 0.24 N. The result indicated that there was a significant difference between the groups, with a t-value of 18.30 and a p-value of 0.01. The retention of ball attachment (Group A) was comparatively higher than that of magnet attachment (Group B). The analogous result was noted by Setz et al. [29] that ball attachment had an increased retention force value of 85 N compared to magnet attachment with a retention force value of 3 N. While Burns et al. [30] and Davis and Packer [31] concluded that both attachment systems, i.e., ball and magnet, showed a statistically significant increase in patient satisfaction compared to the conventional mandibular denture.

Limitations of the study

Even though this study provides valuable insights into how well ball and magnet attachments on mandibular implants retain overdentures, we should acknowledge a few limitations. The small number of participants in the study might limit how widely we can apply these findings. Additionally, the study only looked at two types of attachments, so comparisons with other options are limited. Finally, the long-term performance of these attachments, including wear and maintenance, was not assessed. Future research with more participants, a wider variety of attachments, and longer follow-up periods would give us more comprehensive and practical results.

## Conclusions

In the present study, a comparative evaluation was done to assess the retention of mandibular implant-retained overdentures using two different attachment systems: ball and magnet attachments. The findings revealed that overdentures retained with ball attachments demonstrated greater retention compared to those with magnet attachments. The ball attachment group showed consistently higher retention values, whereas the magnet attachment group exhibited comparatively lower retention. Statistical analysis confirmed that the difference in retention between the two groups was significant. Based on these results, it can be concluded that ball attachments provide superior retention for mandibular implant-retained overdentures when compared to magnet attachments. This suggests that ball attachments may offer better functional stability and patient satisfaction in clinical applications where enhanced denture retention is desired.
